# Quantifying Cost-Effectiveness of Controlling Nosocomial Spread of Antibiotic-Resistant Bacteria: The Case of MRSA

**DOI:** 10.1371/journal.pone.0011562

**Published:** 2010-07-16

**Authors:** Marjan W. M. Wassenberg, G. Ardine de Wit, Ben A. van Hout, Marc J. M. Bonten

**Affiliations:** 1 Department of Internal Medicine and Infectious Diseases, University Medical Center, Utrecht, The Netherlands; 2 Department of Medical Microbiology, University Medical Center, Utrecht, The Netherlands; 3 Julius Center for Health Sciences and Primary Care, University Medical Center, Utrecht, The Netherlands; 4 National Institute of Public Health and the Environment, Bilthoven, The Netherlands; University of Liverpool, United Kingdom

## Abstract

**Background:**

The costs and benefits of controlling nosocomial spread of antibiotic-resistant bacteria are unknown.

**Methods:**

We developed a mathematical algorithm to determine cost-effectiveness of infection control programs and explored the dynamical interactions between different epidemiological variables and cost-effectiveness. The algorithm includes occurrence of nosocomial infections, attributable mortality, costs and efficacy of infection control and how antibiotic-resistant bacteria affect total number of infections: do infections with antibiotic-resistant bacteria replace infections caused by susceptible bacteria (replacement scenario) or occur in addition to them (addition scenario). Methicillin-resistant *Staphylococcus aureus* (MRSA) bacteremia was used for illustration using observational data on *S. aureus* bacteremia (SAB) in our hospital (n = 189 between 2001–2004, all being methicillin-susceptible *S. aureus* [MSSA]).

**Results:**

In the replacement scenario, the costs per life year gained range from € 45,912 to € 6590 for attributable mortality rates ranging from 10% to 50%. Using € 20,000 per life year gained as a threshold, completely preventing MRSA would be cost-effective in the replacement scenario if attributable mortality of MRSA is ≥21%. In the addition scenario, infection control would be cost saving along the entire range of estimates for attributable mortality.

**Conclusions:**

Cost-effectiveness of controlling antibiotic-resistant bacteria is highly sensitive to the interaction between infections caused by resistant and susceptible bacteria (addition or replacement) and attributable mortality. In our setting, controlling MRSA would be cost saving for the addition scenario but would not be cost-effective in the replacement scenario if attributable mortality would be <21%.

## Introduction

Nosocomial infections caused by antibiotic-resistant bacteria, such as methicillin-resistant *Staphylococcus aureus* (MRSA), pose a global health care problem, resulting in direct costs associated with increased health care consumption and indirect costs due to morbidity and loss of life years [1]–[4]. There is considerable geographic variation in incidence of such infections. Reported MRSA rates among *Staphylococcus aureus* bloodstream infections are still <1% in countries with nationwide infection control policies for MRSA, such as in Scandinavian countries and the Netherlands [5], and have been as high as over 50% in countries without such infection control programs (e.g. U.S.A.) [6], [7] or where such measures have been abandoned (e.g. United Kingdom) [8]. Yet, such infection control strategies may affect quality of patient care [9] and their effectiveness has not been unequivocally demonstrated in settings with existing high endemic levels [10].

Costs and benefits of an infection control program are best expressed in universal outcome measures, such as costs per (quality adjusted) life year gained, but to the best of our knowledge, no estimates of life years gained have been reported. In the present study we developed a mathematical algorithm to determine cost-effectiveness of containing infections with antibiotic-resistant nosocomial pathogen. To illustrate its use and explore the importance of several parameters we have applied the algorithm to the MRSA epidemiology in our hospital, which had no case of MRSA bacteremia between 1991 and 2004. The relevant variables in this algorithm include the total number of *S. aureus* bacteremias (SAB, which includes both MRSA and methicillin-susceptible *S. aureus* (MSSA)), the attributable mortality due to infection with a resistant pathogen (in this case MRSA), the costs of infection control, the number of infections prevented (i.e., efficacy) and how antibiotic-resistant bacteria affect the total number of SAB. Using available data from our hospital, and realistic ranges of estimates of the unknown variables, we explored the effect of the epidemiological variables on the costs per life year gained of our control policy.

Of note, a modeling approach always represents a huge simplification of real life and is here used to demonstrate differences across scenarios, rather than to precisely quantify cost-effectiveness of infection control interventions. Hence, our paper is not intended as an economic evaluation of our hospital infection control policies, but merely as an exploration of the dynamic interactions between important epidemiological parameters and cost-effectiveness of infection control policies.

## Methods

### Model design and parameters

A mathematical algorithm was developed (see Appendix S1 for details), that included occurrence of nosocomial SAB, costs and efficacy of infection control, attributable mortality, and the interaction between MRSA and MSSA infection rates. We had accurate estimates of the costs of the MRSA control policy in our hospital [11], its efficacy during this period (being 100%), incidence data of nosocomial SAB, and the characteristics of patients developing nosocomial SAB. There is less certainty, though, about the other variables in the algorithm: the attributable mortality due to MRSA infection and how MRSA affect total number of SAB. Estimates of attributable mortality of MRSA bacteremia, as compared to MSSA, have ranged from 0% to 50% [1]–[3], [12]–[15]. The interaction between MRSA and MSSA infection rates is also largely unknown. Some data suggest that MRSA infections replace MSSA infections with no effects on the total burden of *Staphylococcus aureus* infections (a scenario further referred to as “replacement”) [16], [17]. Other reports suggest that rising incidences of nosocomial MRSA infections add to the incidence of MSSA infections, and that reductions in MRSA infections (through intervention programs) leave MSSA infection rates unaffected [8], [18], [19]. In this scenario (further referred to as “addition”) emergence of MRSA would increase the total burden of *Staphylococcus aureus* infections. Cost-effectiveness was determined for different ranges of the two possible scenarios in which MRSA either results in addition to or replaces cases of nosocomial MSSA bacteremia and when both scenarios simultaneously occur. We assumed that the proportion of MRSA bacteremia among SAB with a less effective infection control strategy, would increase from 1% (as currently in the Netherlands [5]) up to 50% (as witnessed in countries without a control policy [6], [7]). The MRSA control strategy is predominantly based on distancing carriers from non-carriers to prevent exogenous transmission, and was, therefore, assumed not to change MSSA bacteremia rates. Naturally, eradication therapy for MRSA carriage could also reduce MSSA infection rates [20], but this was only applied after hospital discharge.

### Setting

The University Medical Center in Utrecht, The Netherlands, is a public teaching hospital with 1042 beds. Our MRSA policy is based on the Dutch guideline [21] and includes isolation of all patients colonized with MRSA and pre-emptive isolation of all patients with high risks for colonization. In the latter, isolation measures are discontinued when inventory cultures are negative. If MRSA is unexpectedly found in a clinical culture, all contacts (both patients and health care workers) are screened for MRSA colonization. In case of an outbreak a ward may be temporarily closed to new patients to prevent further spread. Eradication therapy for MRSA carriage is only considered after hospital discharge when patients are recovered with absence of wounds, catheters etc. Our prevention strategy was 100% effective in preventing MRSA bacteremia; because numbers of other MRSA infections were negligible, these were not taken into account.

### Data collection

All patients in our hospital with nosocomial SAB (defined as a blood culture with *S. aureus* obtained more than 48 hours after hospital admission) between 1 May 2001 and 30 April 2004 were identified. Demographic and clinical information was, retrospectively, determined from medical charts. The presence of underlying chronic diseases such as chronic obstructive pulmonary disease, diabetes mellitus, chronic renal insufficiency, cancer, chronic cardiac disease and congenital anomalies was recorded. For simplicity, we assumed a dichotomous comorbidity measure in our analyses. Univariate logistic regression analysis was used to determine whether age, gender and comorbidities were associated with survival. Ethical approval was not required for this retrospective case analysis.

### Analyses

Using actual data of SAB incidence and costs of MRSA control in our hospital, we calculated the costs per life year gained with our MRSA policy. The total number of SAB equals the sum of SAB caused by MSSA and MRSA. The difference between observed mortality of SAB in our hospital and the estimated mortality should a proportion of SAB episodes be caused by MRSA, because of a less effective or no MRSA policy, represents the crude number of lives gained with our infection control policy. For those patients that survived SAB in our hospital, average remaining life expectancy was determined using life tables from Statistics Netherlands, which were adjusted for age. We assumed that the presence of comorbidities decreases ones life expectancy with 30% [22], [23]. Effects (life years gained) were discounted at 1.5%, as recommended in the Netherlands [24]. The cost per life year gained were subsequently calculated as the incremental costs for infection prevention and additional length of hospital stay of survivors, divided by the life years gained, for a period of one year of executing Dutch MRSA policy.

### Costs

The cost analysis was performed from a hospital perspective. Costs include costs of infection prevention and incremental costs due to increased length of stay of survivors. The annual costs of MRSA infection control in our hospital were calculated in 2000 [11], being € 277,400, which included costs for microbiological cultures, disposable infection prevention material, cleaning and decontamination, loss of hospitalization days and surgical procedures during outbreaks, temporary suspension of health care workers from work and treatment of MRSA carriers. The costs related to MRSA control in our hospital for the year 2000 have been adapted to 2004 using the consumer price index as collected by Statistics Netherlands and were for simplicity fixed at € 308,533 per year. The costs for additional length of hospital stay attributable to SAB were calculated as the difference between the costs for hospital stay after SAB had been diagnosed with the infection control policy and the costs for hospital stay should SAB have been diagnosed without the infection control policy. The latter estimation was based on expected survival and death rates of patients without infection control and the costs of hospital stay for survivors and non-survivors as observed in the patients in our hospital.

Costs of hospital stay were € 1684 and € 337 for an ICU and non-ICU day, respectively, which are the general cost prices calculated by the Dutch Health Care Insurance Board (CVZ) for 2004 [24]. Costs were expressed in Euros and not discounted, as all costs (infection prevention policy, hospitalization) are made in one year.

### Sensitivity analyses

We explored which uncertain variables were of most influence on the cost-effectiveness in both scenarios (replacement or addition). We have used a distribution of attributable mortality of MRSA bacteremia as compared to MSSA bacteremia, from 0% to 50% [1]–[3], [12]–[15]. We varied the proportion of MRSA bacteremia among SAB with a less effective or no infection control strategy from 10% to 50%. Also we explored the outcome with a less effective MRSA control policy, reducing MRSA prevalence from 50% to 45%, up to 5%. Threshold analyses were performed to determine break-even values for costs per life year gained, i.e., when all costs of infection control plus all the costs related to additional length of stay of surviving patients were compensated by the savings due to less infections. For this threshold analyses, the Dutch informal threshold of € 20,000 per (quality adjusted) life year gained was used.

## Results

### Baseline characteristics

During the 3-year period, there were 189 patients with a nosocomial SAB, all MSSA. 115 (61%) were male and ages of the patients ranged from 0 months to 95 years (mean 39 years) with 48 (25%) infants. 117 (62%) patients had underlying comorbidity of whom 75 (64%), 33 (28%) and 9 (8%) had one, two and three or more chronic conditions, respectively. In 76 (40%) patients MSSA bacteremia resulted from documented infected intravascular devices. The all-cause in-hospital mortality of the patients was 16% (n = 30). Differences between surviving and succumbing patients are presented in Table 1. In logistic regression analysis, age was associated with in-hospital mortality (odds ratio 1.03, 95% confidence interval 1.01–1.05, p = 0.002), the presence of gender and comorbidities appeared non-significant.

**Table 1:** Baseline characteristics of patients with *S. aureus* bacteremias (SAB) by outcome of hospital stay.

Outcome of patients with SAB		
**Survived**		N = 159
	Age, years (SD)	34 (31)
	Life expectancy, undiscounted, adjusted for comorbidity	38
	Length of hospital stay ICU after SAB (SD)	14 (38)
	Length of hospital stay general ward after SAB (SD)	24 (29)
	Incremental costs of hospital stay after SAB (€)	32,225
**Death**		N = 30
	Age, years (SD)	61 (23)
	Life expectancy, undiscounted, adjusted for comorbidity	15
	Length of hospital stay ICU after SAB (SD)	5 (12)
	Length of hospital stay general ward after SAB (SD)	3 (5)
	Incremental costs of hospital stay after SAB (€)	9319

Values are expressed as means.

### Cost-effectiveness in the replacement and addition scenario

**Figure 1:**
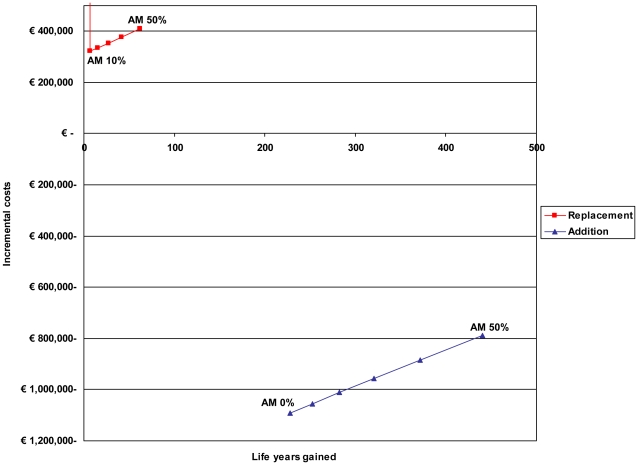
Incremental costs and life years gained per year infection control. In the replacement and addition scenario for estimates of attributable mortality (AM) of MRSA bacteremia, as compared to MSSA, ranging from 0% to 50%.

In the replacement scenario, when the total burden of SAB remains the same over time, while the prevalence of MRSA SAB increases, there will always be incremental costs due to infection control (Figure 1, Table 2). When MRSA would have no attributable mortality there are no life years gained in the replacement scenario and costs per life year gained would be infinite. Considering the Dutch threshold value for cost-effectiveness of € 20,000 per life year gained a MRSA infection control strategy would be cost-effective when attributable mortality of MRSA is ≥21% in the replacement scenario. In contrast, in the addition scenario, the MRSA infection control policy reduces the total absolute numbers of SAB and will always be cost-saving, irrespective of attributable mortality (Figure 1, Table 2). Savings increase with lower attributable mortality rates, as an increasing number of SAB survivors generate higher additional health care costs, compared to the non-survivors.

**Table 2:** Costs, effects and break-even values for different attributable mortality rates in the replacement and addition scenario.

	Attributable mortality					
	0%	10%	20%	30%	40%	50%
**Life years gained, discounted**						
Replacement	0	7.0	15.7	26.8	41.7	62.3
Addition	227.9	252.3	282.6	320.9	371.3	440.3
**Total incremental costs (**€**)**						
Replacement	308,533	321,384	335,305	352,822	377,885	410,557
Addition	−1,092,781	−1,056,128	−1,011,708	−955,961	−884,808	−789,898
**Cost per life year gained per year MRSA policy (**€**)**						
Replacement	NA^b^	45,912	21,357	13,165	9062	6590
Addition	−4795	−4186	−3580	−2979	−2383	−1794
**Break-even values costs infection control policy per year (**€**)** a						
Replacement	NA^b^	127,783	287,257	491,900	764,120	1,144,144
Addition	5,959,726	6,411,444	6,971,136	7,682,879	8,618,626	9,904,560

a Assuming the Dutch threshold value for cost-effectiveness of € 20,000 per life-year gained.

b NA, not applicable.

When the proportion of MRSA bacteremia among SAB without an infection control strategy would be lower than 50%, life years gained are less and costs per life year gained will be higher for both scenarios (data not shown). Threshold analyses for costs of infection control policies to be cost-effective are presented in Table 2.

### Effectiveness of infection control strategies

**Figure 2:**
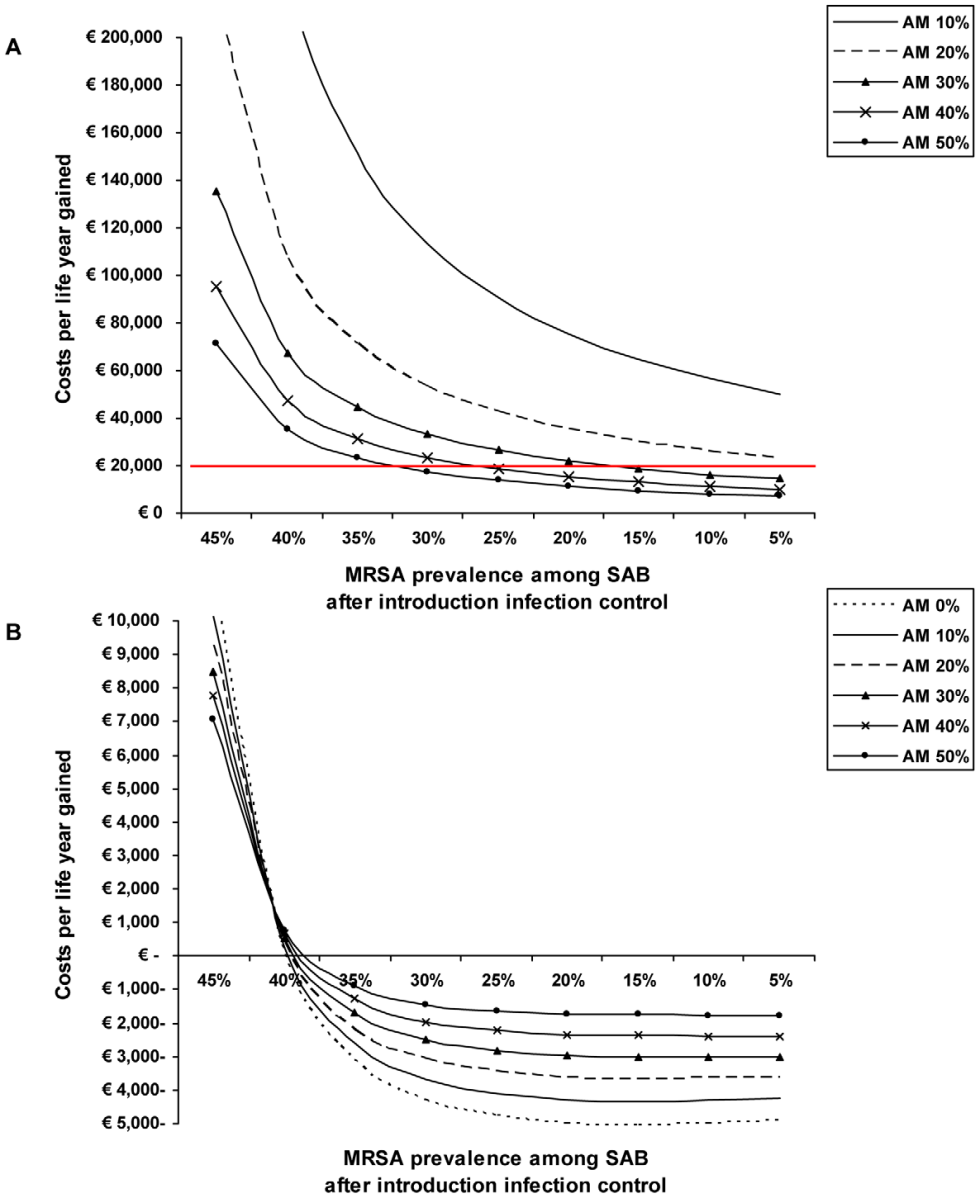
Costs per life year gained as a function of effectiveness of infection control policies and attributable mortality (AM). A) In the replacement scenario. The 20,000 euro line on the vertical axis reflects the Dutch threshold value for cost-effectiveness. B) In the addition scenario.

In Figure 2 we have depicted costs per life year gained as a function of the effectiveness of an infection control policy and attributable mortality. An infection control policy in the replacement scenario will only be cost-effective, assuming the Dutch threshold value for cost-effectiveness of € 20,000 per life year gained, at higher attributable mortality rates and depends on the effectiveness of the infection control strategy (Figure 2a). For example, when the infection control strategy reduces MRSA prevalence from 50% to 30% this will only be cost-effective if attributable mortality is above 45%. If MRSA infections would be additional to MSSA infections infection control strategies would already be cost-neutral (i.e. paid back by additional savings) when the MRSA prevalence is reduced from 50% to 40% with attributable mortality of 0% and will be cost-saving with increasing effectiveness of the MRSA control policy, for all levels of attributable mortality (Figure 2b).

### Mixing replacement and addition scenarios

**Figure 3:**
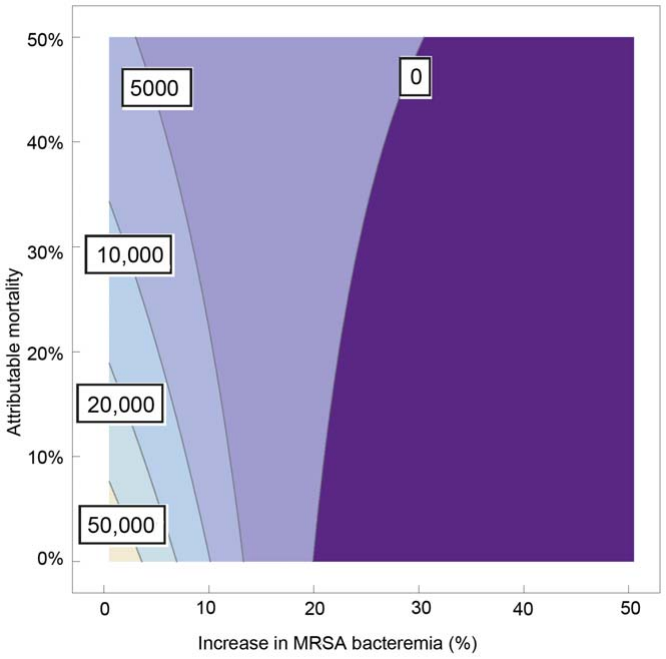
Mixing replacement and addition scenarios. Costs per life year gained as a function of the absolute increase in MRSA bacteremia incidence (horizontal axis) and the rate of attributable mortality of MRSA as compared to methicillin-sensitive *Staphylococcus aureus* (MSSA) (vertical axis) for the scenario that 50% of all *S. aureus* bacteremias cases are caused by MRSA.

Most probably both scenarios occur, and the ultimate effect of an infection control policy will be a combination of absolute reduction and replacement of infections. In Figure 3 we have depicted the costs per life year gained as a function of this interaction (horizontal axis) and the rate of attributable mortality of MRSA as compared to MSSA (vertical axis).

With 100% replacement (no additional cases of SAB), costs per life year gained will be over € 20,000 if attributable mortality is less than 20%. Yet, if attributable mortality is 0%, there should be at least an increase in SAB incidence of 7% to keep costs per life year gained less than € 20,000. And, still without attributable mortality, infection control would become cost-saving if the increase in total SAB is more than 20%.

## Discussion

We have developed an algorithm to determine the cost-effectiveness of controlling nosocomial infections with antibiotic-resistant bacteria, taking important epidemiological variables into account. We conclude that, apart from obvious variables such as costs and effectiveness of infection control, the relative changes in infection incidence rates caused by resistant and (more) susceptible bacteria and the attributable mortality due to infections with resistant bacteria have the largest impact on cost-effectiveness. The latter two variables, though, have not been accurately quantified for most pathogens, which seriously hampers evidence-based decision making for many settings. Based upon recent studies the attributable mortality due to MRSA infections probably is lower than previously assumed [15], [25], and our findings in the lower range of attributable mortality should, therefore, be considered as most realistic. Using the Dutch MRSA control policy as an example we conclude that there is strong evidence that this policy (being almost 100% effective) is cost-effective, and can even be cost-saving from the hospital perspective, under the assumption that MRSA infections would add to the total burden of nosocomial *S. aureus* infections, and not replace current MSSA infections. From a societal perspective, the improved lifespan will balance out the cost of hospital care.

Cost-effectiveness analysis of controlling the spread of antibiotic-resistant bacteria in high endemic settings has been attempted in only few studies. In a French case-control study in an ICU with a 4% admission prevalence of MRSA, an infection control program would be cost-saving compared to the mean costs attributable to MRSA infections as long as the MRSA infection rate was reduced by at least 14% [26]. And in another study in Germany, a MRSA screening program, including pre-emptive contact isolation, was cost-saving if at least 48% of the predicted hospital-acquired MRSA infections would be prevented [27]. The MRSA prevalence in this study was 20.6% in the screened high risk group. Cost-savings achieved by screening and surveillance programs in endemic settings [28]–[31] and during MRSA outbreaks [32], [33] have been reported. However, there has not been a past cost analysis that took the possible scenarios of replacement or addition into account, nor has the cost-effectiveness of a MRSA control policy ever been expressed in costs per life year gained.

As shown in this study, our MRSA control strategy would be cost-effective and even cost-saving if MRSA infections would add to the total number of *S. aureus* infections. However, when using the same parameters for attributable mortality, costs of infection prevention and MRSA prevalence, the same strategy might not be considered cost-effective when MRSA infections would replace nosocomial MSSA infections. Obviously, both scenarios will coexist, excluding the possibility of a “100% additional” or “100% replacement” scenario. Up till now, only few studies provide some quantification of this important parameter. In a recent longitudinal study from the UK overall rates of SAB rose between 1997 and 2004 which was due to an increase in MRSA bacteremia added to a stable MSSA bacteremia rate [8], whereas older studies provided evidence for replacement [16], [17]. Importantly, in all studies only MRSA and MSSA infections were considered. Yet, hospitalized patients are prone to colonization and infection with many bacteria. And although preventing spread of MRSA may reduce the incidence of MRSA infection, these patients are still prone to infection with, for instance, Gram-negative bacteria. Only considering MSSA and MRSA might suggest a reduction in the total number of *S. aureus* infections (supporting the scenario of addition), but might not be associated with a reduction in the total number of nosocomial infections, which would suggest replacement of MRSA by other antibiotic-resistant bacteria. One might argue that the nosocomial infection syndromes caused by *S. aureus* (mainly skin and soft tissue infections, post-surgical wound infections and catheter-related infections) do not overlap to a great extent with those caused by Gram-negative bacteria, and that replacement might be more relevant when reducing infections caused by certain multi-resistant Gram-negative pathogens. Yet, to the best of our knowledge, there are no studies available that have taken all different pathogens into account when determining the cost-effectiveness of infection control programs. With such data, the numbers of MSSA infections as used in our algorithm should simply be replaced by numbers of non-MRSA infections.

Naturally, threshold levels for costs per life year gained for preventive measures are determined by political and societal circumstances and will, therefore, differ per country. With the current accepted costs per life year gained in the Netherlands of € 20,000 [34], [35] our findings suggest that the Dutch infection control policy is cost-effective or even cost-saving along the entire range of estimates for attributable mortality rates for the addition scenario. With the replacement scenario, though, there will always be incremental costs due to infection control, which would be cost-effective (according to Dutch standards) when attributable mortality of MRSA is at least 21%. In our analyses we have used Dutch standards for discounting life years gained, using annual rates of 1.5%. With higher discount rates (3%), which are common in the U.S.A. and the UK, the number of life years gained will be lower resulting in higher costs per life year gained.

Our study has several limitations. First, our example of MRSA control is based on data from a single tertiary care hospital in the Netherlands, and may, therefore, not be fully generalizable to hospitals with different care levels or to settings in other countries. The costs associated with the MRSA policy have been calculated for our hospital and were estimated to be € 308,533 per year between 1991 and 2000 [11]. More recently the costs in another large Dutch teaching hospital have been estimated at € 215,559 per year between 2001 and 2006 [36]. For simplicity, we have used a fixed cost price, though when control measures would be instituted in high endemic settings initial costs may well be higher. Second, for several reasons we based our analysis on bacteremia cases only, as they unequivocally represent infections and have been used for determination of attributable mortality rates. If other nosocomial infections would have been taken into account (e.g. surgical or pulmonary) the results of our study might have been different, depending on the life expectancy and survival rates of patients with these infections. Third, accurately estimating incremental length of hospital stay attributable to SAB is hampered by omitted variable bias and simultaneity bias [37], and our estimates, therefore might be relatively high. However as we used incremental costs for additional length of hospital stay, this did not influence our conclusions.

In conclusion, our study illustrates how cost-effectiveness of controlling nosocomial infections with antibiotic-resistant bacteria can be determined using a mathematical algorithm. Our algorithm may be used in different settings, using local data, to determine cost-effectiveness in other settings.

## Supporting Information

Appendix S1 Supporting information.
(0.07 MB DOC)
