# Supplementary material for: Quantifying Cost-Effectiveness of Controlling Nosocomial Spread of Antibiotic-Resistant Bacteria: The Case of MRSA
Source: PLoS One. 2010 Jul 16;5(7):e11562. doi: 10.1371/journal.pone.0011562 (PMC2905392; doi:10.1371/journal.pone.0011562)
Supplement: Appendix S1 — Supporting information. (0.07 MB DOC) [file pone.0011562.s001.doc]

**Appendix S1**

We use the following notation:

$N^{o}$= number of infected patients without the Dutch infection control policy

$N^{M\_o}$= number of MRSA infected patients without the Dutch infection control policy

$N^{nM\_o}$= number of non-MRSA infected patients without the Dutch infection control policy

$P^{m}$= probability that the infection is MRSA without the Dutch infection control policy

By definition:

$$\begin{matrix} N^{o}=N^{M\_o}+N^{nM\_o} \\ N^{M\_o}=P^{m}.N^{o} \\ N^{nM\_o}=(1-P^{m}).N^{o} \end{matrix}$$

We assume that the Dutch infection control policy has no effect on the number of non-MRSA infections:

$$N^{nM\_nl}=N^{nM\_o}$$

**Addition scenario:**

The infection control policy may prevent the incidence of new MRSA infections; this may be expressed as:

$$N^{M\_nl}=(1-\varepsilon_{i}).N^{M\_o}$$

$$N^{o}-N^{nl}=\varepsilon_{i}.N^{M\_o}=\varepsilon_{i}.P^{m}.N^{o}$$

The expected number of infections without the infection control policy can be written as:

$$N^{0}=\frac{N^{nl}}{(1-\varepsilon_{i}P^{m})}$$

$\varepsilon_{i}$measures the relative effectiveness of the infection control policy in terms of incident SAB infections.

$$N^{M\_o}=P^{m}N^{o}=\frac{P^{m}.N^{nl}}{(1-\varepsilon_{i}.P^{m})}$$

**Replacement scenario:**

The infection control policy may prevent prevalent infections to be of the MRSA-type and shift infections from MRSA to non-MRSA. The mortality due to MRSA-infections is higher than those of non-MRSA infections.

We note:

$N_{d}^{o}$= the number of people dying without the infection control policy:

$N_{d}^{nl}$= the number of people dying with infection control policy:

$P_{d}^{M}$= the probability to die of an MRSA-infection

$P_{d}^{NM}$= the probability to die of a non-MRSA-infection

We define the difference between the probability to die of a MRSA-infection and the probability to die of a non-MRSA-infection as:

$$P_{d}^{nM}=(1-\delta).P_{d}^{M}$$

Without the infection control policy, the number of patients dying is:

$$N_{d}^{o}=P_{d}^{M}N^{M\_o}+P_{d}^{nM}N^{nM\_o}$$

or:

$$N_{d}^{o}=\left(P_{d}^{M}.P^{m}+(1-\delta)P_{d}^{M}(1-P^{m})\right).\left(\frac{N^{nl}}{(1-\varepsilon_{i}.P^{m})}\right)=P_{d}^{M}\left(1-\delta(1-P^{m})\right).\left(\frac{N^{nl}}{(1-\varepsilon_{i}.P^{m})}\right)$$

With the infection control policy, and taking account of the shift of infections towards non-MRSA, we write

$$N_{d}^{NL}=P_{d}^{M}N^{M\_nl}.(1-\varepsilon_{p})+P_{d}^{nM}\left(N^{nM}+N{}^{M\_nl}.\varepsilon_{p}\right)$$

$\varepsilon_{p}$is the % decrease in the infections being of the MRSA type.

To calculate the number of patients dying without the infection control policy, given estimates of the effectiveness of the policy we write:

$$\begin{matrix} N_{d}^{NL}=P_{d}^{M}.N^{o}.\left((1-\delta)+P^{m}.\left((1-\varepsilon_{i}).(1-\varepsilon_{p})-(1-\delta).\left(1-(1-\varepsilon_{i})(\varepsilon_{p})\right)\right)\right)\Rightarrow\\ P_{d}^{M}=\frac{N_{d}^{nl}}{N^{nl}}.\frac{(1-\varepsilon_{i}P^{m})}{\left((1-\delta)+P^{m}.\left((1-\varepsilon_{i}).(1-\varepsilon_{p})-(1-\delta).\left(1-(1-\varepsilon_{i})(\varepsilon_{p})\right)\right)\right)} \end{matrix}$$

$$N_{d}^{o}=P_{d}^{M}\left(1-\delta(1-P^{m})\right).\left(\frac{N^{nl}}{(1-\varepsilon_{i}.P^{m})}\right)$$

This calculation neglects that it is especially the elderly with an increased risk to die of an infection during their hospital stay. And indeed, a logistic regression on the Dutch data learns that there is a significant effect of age (the presence of co-morbidities and gender appear non-significant) on in-hospital mortality. This implies that it is highly likely that the gain in life years gained is over-estimated if this is not taken into account.

So, we find that:

$$P_{d}(age)=\frac{1}{1+\exp(-3.205+0.032.age)}$$

This relationship does not distinguish between patients with and without MRSA.

We may predict the number of patients dying in the Netherlands as:

$$N_{d}^{nl}=\int_{age} \left(1-\frac{1}{1+\exp(-3.205+0.032.age)}\right)d\left(f\left(age\right)\right)$$

Assuming that prevention, in terms of both the shift as well as the incidence does not affect different ages differently, we may use earlier results to calculate a risk ratio due to the infection control policy as:

$$RR=\frac{\ln\left(1-\frac{N_{d}^{o}}{N_{o}}\right)}{\ln\left(1-\frac{N_{d}^{nl}}{N_{nl}}\right)}$$

We may estimate the number of deaths without an infection control policy corrected for the age distribution as:

$$N_{d}^{o}=\int_{age} 1-\exp\left(RR*\ln\left(\frac{1}{1+\exp(-3.205+0.032.age)}\right)\right)d\left(f\left(age\right)\right).\frac{N^{o}}{N^{nl}}$$
